# Thalassemia-Associated mixed hypogonadism (TAMH): unraveling a unique endocrine pattern and its impact on cardiovascular risk

**DOI:** 10.1007/s12020-025-04432-3

**Published:** 2025-09-20

**Authors:** Maddalena Casale, Martina Errico, Francesca Allosso, Lucia Digitale Selvaggio, Graziella Grande, Claudia Di Ludovico, Raffaele Navarra, Domenico Roberti, Maria Maddalena Marrapodi, Silverio Perrotta, Daniela Pasquali

**Affiliations:** 1 Department of Woman, Child and General and Specialized Surgery, University of Campania L. Vanvitelli, Naples, Italy; 2 Department of Advanced Medical and Surgical Sciences, University of Campania L. Vanvitelli, Naples, Italy

**Keywords:** Transfusion-dependent thalassemia, Hypogonadism, Cardiovascular risk, Thalassemia-associated mixed hypogonadism, Iron overload.

## Abstract

**Background:**

Transfusion Dependent Thalassemia (TDT) is a hereditary hematologic disorder requiring chronic transfusions, leading to iron overload and frequent endocrinological complications, notably hypogonadism, defined as secondary or hypogonatropic, potentially impacting quality of life and cardiovascular (CV) risk. This study aimed to evaluate the pattern of hypogonadism in TDT and its impact on CV risk compared to non-hypogonadal subjects with TDT and hypogonadal subjects without TDT.

**Methods:**

A cross-sectional observational study assessed 22 TDT patients with hypogonadism (H-TDT) and 31 TDT without a diagnosis of hypogonadism (NH-TDT) aged 18 years or older. A control group of adult non-thalassemic hypogonadotropic hypogonadism patients (HH-C) was included for comparison. Data on medical history, clinical signs/symptoms of hypogonadism, lifestyle factors, blood pressure, and hormone levels were collected. CV risk was assessed using Framingham and Progetto Cuore scores, as well as echocardiographic parameters.

**Results:**

H-TDT patients had significantly higher FSH and LH levels than HH-C (*p* < 0.001). Significant differences in CV and total cholesterol levels were found between TDT patients with and without hypogonadism (*p* = 0.046, *p* = 0.004, respectively). CV risk was similar between H-TDT patients and the HH-C.

**Conclusions:**

The nature of hypogonadism in TDT is essentially mixed, definitively transcending a purely hypogonadotropic profile. Furthermore, while the CV risk is higher in subjects with H-TDT compared to NH-TDT, it remains extraordinarily low and not pathological according to two validated scores in the general population. This suggests a need to identify more suitable tools for the CV risk in TDT patients.

## Introduction

Transfusion Dependent Thalassemia (TDT) is an inherited hematological disorder requiring regular blood transfusions and chronic iron chelation therapy to manage severe iron accumulation as high as 0.3–0.5 mg/kg/day [1–9]. Endocrine glands are particularly susceptible to iron toxicity, and endocrinopathies are the most frequent disease-related complications nowadays [10–14]. Hypogonadism stands out as a particularly prevalent complication, with studies reporting a prevalence that can vary between 40% and 80% depending on the studied populations [13–18].

The pathogenesis of hypogonadism in these patients is significantly linked to iron overload and is reported to be primarily due to the accumulation of iron in the pituitary gland. This iron deposition can damage the gonadotropic cells of the pituitary, leading to central defects of the hypothalamus or pituitary, resulting in secondary hypogonadism or hypogonadotropic hypogonadism (HH). Iron deposition in the anterior pituitary gland has been demonstrated from the first decade of life, but clinical signs and symptoms do not occur until the onset of puberty, with delayed puberty development. In adulthood, there may be an asymptomatic phase of pituitary siderosis before hypogonadism occurs. Subsequently, gonadotropin reserve decreases significantly, with a reduction in pulsatile gonadotropin activity that can lead to irreversible axis damage. In males with TDT, hypogonadism can manifest with reduced testosterone levels and altered secondary sexual characteristics, including gynecomastia, decreased libido, and easy fatigability. Hypogonadism itself may influence cardiovascular health [19, 20]. A low testosterone level at pubertal age is a strong indicator of mixed hypogonadism. Hypogonadism in female TDT patients can manifest in delayed or absent sexual development, menstrual irregularities with anovulation, infertility, and fatigue [21].

Generally, in TDT patients, hypogonadism is defined as hypogonadotropic [17, 18, 22]. However, severe iron overload can lead to direct damage to the gonads, and hypogonadism could be related to both testicular and pituitary damage. Data of histology of testicular tissue in TDT patients with severe iron overload reveal testicular interstitial fibrosis with small, heavily pigmented, undifferentiated seminiferous tubules and absence of Leydig cells [22]. This direct gonadal damage can impair spermatogenesis and the production of testosterone [23–27]. Studies have reported a significant prevalence of hypogonadism in females with thalassemia, though the exact percentage can vary between 45.8% and 76% [13, 14, 24–26]. The iron overload, however, has a less significant impact in female TDT patients, as the ovarian reserve is preserved in most of them, even in those suffering from amenorrhea.

Low testosterone levels are strongly associated with an increased risk of developing various metabolic disorders. This typically manifests as an increase in both subcutaneous and visceral fat mass, as well as marked insulin resistance and a higher risk of type 2 diabetes mellitus in men affected by hypogonadotropic hypogonadism [28, 29]. Testosterone deficiency adversely affects vascular health and constitutes a risk factor for future cardiovascular events, primarily due to these metabolic alterations, which are key pathophysiological contributors to the development of cardiovascular disease. Testosterone therapy is considered safe from a cardiovascular standpoint when prescribed appropriately, with its potential benefits outweighing the risks [30].

Regarding estrogens, the relationship between their deficiency and increased cardiovascular risk is well established. Women experiencing menopause at earlier ages have a significantly higher risk of developing coronary heart disease, stroke, heart failure, type 2 diabetes, and dyslipidemia compared to those undergoing menopause after 45. This increased risk is largely due to early estrogen deficiency, which normally exerts protective effects on the cardiovascular system by supporting vascular function and regulating glucose and lipid metabolism [31, 32].

This study aimed to verify the pattern of hypogonadism in TDT patients (H-TDT) aged over 18 years, to identify clinical signs and symptoms associated with this condition. Furthermore, the study sought to compare this population with TDT patients without hypogonadism (NH-TDT) and an adult non-thalassemic hypogonadotropic population (HH-C) and to evaluate the differences in metabolic profile and CV risk between TDT patients with and without hypogonadism.

## Methods

### Study population

We performed a cross-sectional observational study. The study population comprised 53 patients diagnosed with TDT, ranging in age from 21 to 75 years, with a mean age of 39.80 years. Among them, 22 TDT patients had hypogonadism (H-TDT). For comparative analysis, 31 TDT without diagnosis of hypogonadism (NH-TDT) and 29 patients with HH-C were also enrolled. These control patients were older than 18 years with a mean age of 38 years. In the control group, 15 patients were affected by congenital hypogonadism, 1 patient by Langerhans cell histiocytosis, 3 patients with morphological alterations of the pituitary region (2 with empty sella and 1 with pituitary hypoplasia), and 10 patients after hypothalamic-pituitary surgery. Selected subjects provided written informed consent after receiving a full description of the study, and they could ask questions. An ethical committee reviewed the investigation protocol, according to the updated version of the Declaration of Helsinki Clinical Trial: NCT01874405; CE Seconda Università degli Studi di Napoli – Azienda Ospedaliera Universitaria SUN – AORN “Ospedali dei Colli” no. 916 (28/07/2016).

Inclusion criteria. All patients with TDT referred to in the Rare Endocrine Diseases Program, AOU Vanvitelli, were included in the study.

Exclusion criteria. Patients were excluded from the study if they were younger than 18 years old, had a history suggesting other causes of hypogonadism rather than thalassemia (history of trauma to the gonads or the head, radiation, drugs known to cause hypogonadism), or had undergone bone marrow transplantation.

Female hypogonadism was defined according to established guidelines as estradiol concentration persistently below 200 pmol/L in the context of amenorrhea of at least 3 months’ duration [33].

Male hypogonadism was defined according to established guidelines as total testosterone levels below 8 nmol/L (230 ng/dL) with physical and psychological symptoms, such as loss of energy, low mood, fatigue, and sexual symptoms [34].

### Assays

Serum was collected in the morning, in the same standard sampling tubes with separator gel. Basic biochemical and hormonal assays performed included the following, with normal reference range reported: a complete blood count, alanine transaminase (5–33 U/L), aspartate aminotransferase (5–32 U/L), urea (10–50 mg/dl), creatinine (0.51–0.9 mg/dl), thyroid stimulating hormone (TSH) (0.4–4.0 microUI/ml, triiodothyronine (1.5–5.9 pg/ml, thyroxine (5.2–15.8 pg/ml), serum calcium (8.6–10.2 mg/dl), serum phosphorus (2.7–4.5 mg/dl), total cholesterol (60–200 mg/dl), HDL-cholesterol (> 45 mg/dl), LDL-cholesterol (10–129 mg/dl) triglycerides (20–175 mg/dl), glycemia (70–100 mg/dl), luteinizing hormone (LH) (for female patients 1.9–9.2 UI/L, for male 2.8–6.8 UI/L), follicle-stimulating hormone (FSH) (for female patients 3.5-9-2 UI/L, for male 1.3–11.8 UI/L), estradiol in females, and testosterone in males. Samples, blinded to identification, were immediately sent to the Laboratory Facility of the AOU Vanvitelli and were evaluated in duplicate: FSH, LH, and TSH were determined by IRMA, using commercial kits [35]. Serum levels of total testosterone were measured using an automated immunoassay (DiaSorin Liaison^®^, Saluggia, VC, Italy) [31]. The normal reference range for total testosterone was 6.77–31.06 nmol/L. Serum levels of estradiol were measured during follicular phase using an automated immunoassay (DiaSorin Liaison^®^, Saluggia, VC, Italy) [36]. The normal reference range was 31–771 pmol/L.

Diagnostic assessment in thalassemic patients was conducted before the initiation of therapy. Following the diagnosis of hypogonadism, all affected patients were prescribed hormone replacement therapy, with adherence varying among individuals. Adherence to hormonal therapy was assessed using self-report questionnaires and patient interviews.

### Cardiovascular risk

Cardiovascular risk was assessed using the Framingham Risk Score and CUORE123 score [37, 38]; in addition, ECG abnormalities and echocardiographic alterations (atrial dilation, diastolic dysfunction, left ventricular hypertrophy) were evaluated.

### Electrocardiographic and echocardiographic measurements

All subjects underwent a routine standard 12-lead body surface ECG recorded at a paper speed of 50 mm/s and gain of 10 mm/mV in the supine position and were breathing freely but not allowed to speak during the ECG recording. In each electrocardiogram lead, the analysis included three consecutive heart cycles wherever possible. ECG with measurable P-waves in less than ten leads were excluded from analysis.

Images were gathered with a standard ultrasound machine (GE Vivid 9, USA) with a 3.5–4 MHz phased array probe (M3S). Left ventricular (LV) diameter and wall thickness were measured from the two-dimensional targeted M-mode echocardiographic tracings in the parasternal short axis to evaluate the presence of ventricular hypertrophy. Ejection fraction was measured using a modified Simpson’s biplane method. Pulsed wave Doppler examination was performed to obtain the following indexes of LV diastolic function: peak mitral inflow velocities at early (E) and late (A) diastole and E/A ratio to evaluate the presence of diastolic dysfunction. Left atrial size was determined by M-mode in the parasternal long axis projection; cavity area of both atria was measured planimetrically in the apical four-chamber view (2D) to evaluate the presence of atrial dilatation [39].

### Data collection and analysis

Data were collected from patient medical records, which were then entered and coded using Microsoft Excel version 2024. The statistical analysis was performed using the Statistical Package for the Social Sciences (SPSS) version 26.

For the analysis, continuous variables were compared using several statistical tests, including Fisher’s exact test, Wilcoxon rank sum test, Pearson’s Chi-squared test, and Wilcoxon rank sum exact test.

The normality of data distribution was assessed using the Shapiro-Wilk test. Each variable’s distribution was examined within groups to verify adherence to the normality assumption. No multivariate analyses were performed, as the primary aim was to compare individual variables between groups. For inferential statistics, continuous variables with a non-normal distribution were analyzed using non-parametric tests, specifically the Wilcoxon rank sum test and the Wilcoxon rank sum exact test. Categorical variables were analyzed with Fisher’s exact test or Pearson’s Chi-squared test, as appropriate. Qualitative data were presented as frequencies and percentages. A p-value of < 0.05 was considered statistically significant for all analyses.

## Results

### Prevalence and clinical manifestations of hypogonadism in TDT

Among the 53 TDT patients enrolled in the study, 22 patients (41.5%) presented with hypogonadism. These included 8 males (36%) and 14 females (64%) (Table 1). All 22 patients with hypogonadism exhibited clinical manifestations. Specifically, 4 women presented with primary amenorrhea and 10 with secondary amenorrhea. Primary amenorrhea was an anamnestic finding at the time of clinical assessment. The four women were between 22 and 35 years of age and had consistently declined hormone replacement therapy. The women included in the study were under 45 years of age and pre-menopausal. In males, the clinical manifestations varied and included asthenia, decreased libido, erectile dysfunction, osteoporosis, and reduced muscle mass.

**Table 1:** Characteristics of the population examined

Characteristics Mean (SD) or *N* (%)	NH-TDT	H-TDT	HH-C	*p*-value^1^	*p*-value^2^
N.	31	22	29		
Age (years)	37 (12)	41 (9)	38 (20)	0.2	0.1
F	17 (55%)	14 (64%)	9 (31%)	0.5	0.02
M	14 (45%)	8 (36%)	20 (69%)		
Compliance to therapy	-	17 (77%)	28 (97%)	-	0.073
Diabetes	0 (0%)	3 (14%)	0 (0%)	0.066	0.074
Smoking	4 (13%)	8 (36%)	7 (25%)	0.5	0.4
Splenectomy	12 (39%)	17 (77%)	-	0.005*	-

The table summarizes the main clinical and demographic features of the three patient groups: non-hypogonadal thalassemic patients (NH-TDT), hypogonadal thalassemic patients (H-TDT), and hypogonadal non-thalassemic patients (HH-C). For each group, data are presented regarding mean age, sex distribution, and the presence of diabetes and smoking habits. In hypogonadal patients (H-TDT and HH-C), adherence to hormone replacement therapy was also assessed. The presence of splenectomy was recorded only in thalassemic patients (TDT and H-TDT). Continuous variables are expressed as mean (standard deviation), and categorical variables as number (percentage).

Age, a continuous variable, was analyzed using the Wilcoxon rank sum test due to non-normality. Categorical variables were compared using Pearson’s chi-squared test, except when low frequencies were present, in which case Fisher’s exact test was applied.

#### Hormone levels

In females, the mean estradiol level measured before starting therapy was 69.75 pmol/L in the hypogonadal group compared to 209.25 pmol/L in the non-hypogonadal TDT patients (NH-TDT). In males, the mean testosterone level measured before starting therapy was 5.66 nmol/L in the hypogonadal group versus 19.78 nmol/L in the NH-TDT patients, a statistically significant difference (*p* < 0.001). The mean FSH and LH levels in the H-TDT population were 4.2 IU/L and 2.8 IU/L, respectively, while in the NH-TDT population, the mean levels were 12 IU/L and 6.4 IU/L, respectively. The differences in both gonadotropin levels between the two TDT groups were statistically significant (*p* < 0.001) (Table 2).

**Table 2:** Hormonal assessment

Hormonal levels Mean (SD)	NH-TDT	H-TDT	HH-C	*p*-value^1^	*p*-value^2^
Estradiol Female (pmol/l)	209.25 (168.87)	69.75 (58.74)	37.08 (7.23)	0.005*	0.7
Testosterone Male (nmol/l)	19.78 (6.35)	5.66 (2.71)	1.98 (0.86)	< 0.001*	0.01*
FSH (IU/L)	12 (17.0)	4.2 (4.0)	1.8 (2.3)	0.006*	0.003*
LH (IU/L)	6.4 (4.7)	2.8 (2.9)	0.9 (1.1)	< 0.001*	0.001*

The table presents serum levels of LH and FSH in all patients, as well as estradiol levels in women and testosterone levels in men in NH-TDT, H-TDT, and HH-C groups. Hormone values are expressed as mean (standard deviation). Variables were analyzed using the Wilcoxon rank sum test. When sample sizes were small or distributions included tied values, the exact version of the test was applied

#### Lipid profile

A significant difference was found in total cholesterol levels (*p* = 0.004) between H-TDT and NH-TDT. The H-TDT group had significantly higher total cholesterol levels compared to the NH-TDT group (Table 3).

#### Splenectomy

Another statistically significant difference (*p* < 0.005) between the two groups pertains to the prevalence of splenectomy; specifically, 77% of H-TDT patients had undergone splenectomy, compared to only 39% of NH-TDT patients.

**Table 3:** CV risk and lipid profile

CV risk and metabolic profile Mean (SD) or *N* (%)	NH-TDT	H-TDT	HH-C	*p*-value^1^	*p*-value^2^
Progetto Cuore score^3^	0.77 (1.04)	0.87 (0.83)	4.12 (7.86)	0.13	0.6
Framingham score^4^	3.2 (3.5)	4.0 (2.9)	8.0 (12.0)	0.046*	0.2
Total cholesterol (mg/dl)	113 (26)	136 (27)	185 (43)	0.004*	< 0.001*
HDL-c (mg/dl)	35 (14)	39 (11)	51 (13)	0.15	< 0.001*
Triglycerides (mg/dl)	93 (42)	106 (62.1)	118 (58.6)	0.907	0.446
LDL-c (mg/dl)	66 (23.8)	78 (32.3)	107 (38)	0.116	0.007*
PAs (mmHg)	112 (10)	107 (9)	119 (9)	0.083	< 0.001*
PAd (mmHg)	61 (7.7)	61 (9.9)	74 (10.2)	0.814	< 0.001*

^1^p-value NH-TDT vs. H-TDT

^2^ p-value H-TDT vs. HH-C

^3^ Low risk < 3%; intermediate risk 3–20%; high risk > 20%

^4^ Low risk < 10%; intermediate risk 10–19%; high risk > 20%

The table presents total cholesterol, HDL cholesterol, LDL cholesterol, triglycerides, systolic blood pressure (PAs), diastolic blood pressure (PAd) and CV risk scores calculated using the progetto Cuore and Framingham models in NH-TDT, H-TDT, and HH-C groups. All values are reported as mean (standard deviation). Variables were analyzed using the Wilcoxon rank sum test. When sample sizes were small or distributions included tied values, the exact version of the test was applied

### Treatment of hypogonadism

All hypogonadal male TDT patients were on replacement therapy with various testosterone formulations, showing good hormonal compensation and improvement in asthenia and libido over time. Among the hypogonadal female TDT patients, only 6 (40%) were on estrogen-progestin replacement therapy, while 8 (60%) refused treatment, indicating poor compliance.

### Cardiovascular risk assessment

The CV risk, assessed using both the Framingham score and the Progetto Cuore score, was low in both the H-TDT and NH-TDT patient groups. A significant difference was observed in the Framingham Risk Score (*p* = 0.046) between H-TDT and NH-TDT. While the mean Framingham risk score appears slightly higher in the H-TDT group, there is considerable overlap in the distributions. Hypertension was not observed in any of the patients, and none were undergoing antihypertensive therapy.

### Echocardiographic evaluation in TDT

In the comparison between the H-TDT group and the NH-TDT group, Ventricular Hypertrophy: 2 (9.1%) patients in the H-TDT group presented with left ventricular hypertrophy, compared to 0 (0%) in the non-H-TDT group. The difference was not statistically significant (*p* = 0.2). Diastolic Dysfunction: 2 (9.1%) patients in the H-TDT group presented with diastolic dysfunction, compared to 4 (13%) in the NH-TDT group. The difference was not statistically significant (*p* > 0.9). Atrial Dilatation: 4 (18%) patients in the H-TDT group presented with atrial dilatation, compared to 6 (19%) in the NH-TDT group. The difference was not statistically significant (*p* > 0.9). Electrocardiogram (ECG) Anomalies: 8 (36%) patients in the H-TDT group had abnormal ECG findings, compared to 12 (39%) in the NH-TDT group. This difference was also not statistically significant (*p* > 0.9).

In summary, the echocardiographic evaluation did not reveal any statistically significant differences in the prevalence of left ventricular hypertrophy, diastolic dysfunction, atrial dilatation, or ECG anomalies between the TDT patients with hypogonadism and those without.

Concerning pre-existing cardiovascular conditions, three patients in the H-TDT group and five in the NH-TDT were affected by cardiomyopathy due to the underlying disease. Cardiovascular history in the NH-TDT group included: one patient had a history of ischemic stroke and congestive heart failure, while two other patients had experienced a single documented episode of atrial fibrillation. No patient in the H-TDT group reported a history of cardiovascular events.

### Comparison between H-TDT and HH-C control group

When comparing the H-TDT patients with the HH-C, there was a significant difference in gender distribution, with a higher prevalence of males in the control group (69% vs. 36%, *p* = 0.02). There were no significant differences in estrogen levels between the female populations. However, the pre-therapy total testosterone levels in males were significantly higher in the H-TDT group (5.66 nmol/L) compared to the HH-C (1.98 nmol/l, *p* = 0.01). Similarly, the mean FSH and LH levels were significantly higher in the H-TDT group (FSH 4.2 IU/L, LH 2.8 IU/L) compared to the HH-C group (FSH 1.81 IU/L, *p* = 0.003; LH 0.91 IU/L, *p* = 0.001). No significant differences were found in the prevalence of smoking or diabetes mellitus between the two groups. Mean total cholesterol, HDL cholesterol levels and LDL cholesterol levels were significantly lower in the H-TDT patients (total cholesterol 136 mg/dL vs. 185 mg/dL, *p* < 0.001; HDL cholesterol 39 mg/dL vs. 51 mg/dL, *p* = 0.002; LDL cholesterol 78 mg/dl vs. 107 mg/dl, *p* = 0.007). The mean systolic and diastolic blood pressure were also significantly lower in the H-TDT group (107 mmHg vs. 119 mmHg, *p* < 0.001; 61 mmHg vs. 64 mmHg, *p* < 0.001). Hypertension was not observed in any of the H-TDT, and none were receiving antihypertensive therapy. Three patients in the HH-C group were affected by essential hypertension and were undergoing pharmacological treatment, but no patients reported a history of cardiovascular events. There were no significant differences in CV risk between the H-TDT patients and the HH-C control group. The calculated risk was generally below 10% according to the Framingham score and below 5% according to the Progetto Cuore score for both groups.

## Discussion

The findings of this single-center study confirm the high incidence of hypogonadism in patients with TDT and suggest a concomitant peculiar CV risk in this population. Low testosterone levels are linked to obesity, metabolic syndrome, type 2 diabetes mellitus, and abnormal lipid profiles, which all contribute to an increased risk of cardiovascular disease. Therefore, testosterone deficiency has a negative impact on men’s vascular health, quality of life and can lead to higher mortality rates [40]. These observations align with the well-established understanding that hypogonadism is a frequent endocrinological complication in TDT, primarily attributed to excessive iron deposition in the pituitary and gonadal area due to chronic transfusions. As many as 51% to 80% of TDT patients may experience pubertal failure, sexual dysfunction, and/or infertility due to hypogonadism. A key finding of our study is that H-TDT patients displayed gonadotropin levels higher than HH-C, suggesting a mixed pattern of systemic damage contributing to hypogonadism in thalassemia. This supports the notion that in thalassemia, hypogonadism might not solely be due to pituitary iron overload (hypogonadotropic hypogonadism) but could also involve direct gonadal damage from iron deposition, leading to a more complex endocrine picture. Histological evidence from Vincenzo De Sanctis et al. [23] shows testicular interstitial fibrosis with iron pigment and absence of Leydig cells in a young adult TDT patient with severe iron overload, indicating direct gonadal damage. This “intermediate clinical entity” of hypogonadism in thalassemia, as characterized by the study, necessitates tailored diagnostic, therapeutic, and follow-up approaches. Finally, hypogonadism in TDT must be considered a mixed defect, involving both primary (gonadal) and secondary (central) components, with iron overload being the primary underlying cause. In conclusion, the diagnostic classification as ‘hypogonadotropic hypogonadism’ should be reevaluated, as it does not encompass the unique characteristics of these patients.

We highlight the difference in CV risk factor profiles between H-TDT and HH-C control group, revealing lower cholesterol levels and systolic blood pressure in the H-TDT group despite comparable overall risk scores, suggesting a complex interplay of factors in thalassemia. The study’s objective to evaluate hypogonadism in TDT patients and analyze their CV risk profile is consistent with the broader literature. Previous studies have explored the interplay between traditional CV risk factors and hypogonadism in influencing cardiac outcomes in these patients, who are living longer due to improved therapies. By analyzing data from 159 patients with extended follow-up, the researchers showed that TDT presents a unique high-risk cardiovascular condition that requires tailored monitoring beyond standard risk assessments. Barbero et al. [41] also emphasize the significant role of hypogonadism in the cardiovascular outcomes of TDT patients, suggesting a specific metabolic risk profile in which hypogonadism plays a relevant part. While no significant difference in overall CV risk scores between H-TDT and HH-C control groups was found, our study directly links hypogonadism with higher CV risk within the TDT population. This could be attributed to the long-term effects of hypogonadism on metabolic parameters and endothelial function, as suggested by Auerbach and Khera, though this specific mechanism would require further investigation within the context of thalassemia [42]. To complement traditional risk scores, our study conducted a comprehensive echocardiographic and electrocardiographic evaluation, focusing on parameters like left ventricular hypertrophy, diastolic dysfunction, atrial dilatation, and ECG anomalies as key indicators of cardiac health and CV risk in TDT. While we found no statistically significant differences in the prevalence of these specific abnormalities between the H-TDT and NH-TDT groups, this finding is critical, considering that conventional Framingham and Progetto Cuore scores indicated an extraordinarily low overall cardiovascular risk. This discrepancy highlights that traditional tools may underestimate the true cardiovascular risk in TDT patients due to their unique metabolic profile and low blood pressure. Therefore, our detailed cardiac assessment highlights the complex, multifactorial nature of CV risk in TDT, reinforcing the urgent need for more tailored monitoring tools beyond standard assessments to capture the unique vulnerabilities of this population.

Furthermore, a meta-analysis focusing on the management of the aging population of Italian patients with TDT highlights the increasing prevalence of endocrine disorders, including hypogonadism, in the aging thalassemia population [43]. The need for vigilant clinical evaluation and appropriate hormonal evaluation, as previously recommended [23, 44], is underscored by the findings of our study, emphasizing the importance of early detection and management of hypogonadism to potentially mitigate long-term complications, including cardiovascular issues.

In TDT, chronic anemia is associated with vasodilated circulation, and blood pressure is significantly lower than in non-thalassemic subjects [45]. The lipid profile differences observed in patients with TDT, and hypogonadism are complex and present some contrasting findings. The study by Barbero et al. noted that low serum lipid levels with low HDL levels were usually observed in their cohort of TDT patients (49.6% had HDL < 40 mg/dL). Interestingly, they also found that patients with hypogonadism in their study had statistically significantly higher levels of total cholesterol and LDL. This aligns with our findings.

Similarly, we showed lower mean total cholesterol (136 mg/dL vs. 185 mg/dL, *p* < 0.001), HDL cholesterol (39 mg/dL vs. 51 mg/dL, *p* = 0.002) and LDL cholesterol (78 mg/dl vs. 107 mg/dl, *p* = 0.007) in the H-TDT compared to HH-C control group, revealing the peculiar metabolic profile in TDT. These discrepancies highlight the complex relationship between thalassemia, hypogonadism, and lipid metabolism. Splenectomy has been associated to alterations in lipid metabolism [46], higher rate of disease-related complications [47], iron-related complications [48] and specific immune dysfunction [49], thus suggesting it is a marker of the severity of the underlying disease [50]. The higher rate of splenectomy in H-TDT patients aligns with these observations.

Thalassemia itself is associated with altered lipid metabolism. Chronic anemia, iron overload, and increased erythropoiesis can affect lipid synthesis and clearance. Hypogonadism, depending on its etiology and the specific hormonal deficiencies, can also independently impact lipid profiles. The combination of these two conditions might lead to complex and sometimes opposing effects on lipid levels. Despite the high prevalence of hypogonadism, some studies using traditional risk scores like Framingham and Progetto Cuore found a low overall CV risk in TDT patients, even those with hypogonadism. However, considering the peculiarity of the TDT lipid profile and baseline low blood pressure, these scores may underestimate the real CV risk in this population.

TDT is a rare disease, and the small sample size represents a limitation that needs to be overcome by larger multicenter studies. The cross-sectional observational design affected causal inference and limited the availability of specific laboratory and/or radiological (e.g. testicular ultrasound, MRI) assessments potentially correlated with hypogonadism and CV risk in our study population. Further studies are needed to include further predictive factors. The greatest strength of our study lies in the presence of a control group affected by a purely central pathology, which allowed us to better define the characteristics of hypogonadism in patients with TDT and the systemic toxic effect of iron. In conclusion, this study reinforces the significant burden of hypogonadism in TDT and its potential association with increased CV risk. The observation of higher gonadotropin levels in TDT with hypogonadism compared to non-thalassemic hypogonadism highlights the unique pathophysiology of endocrine dysfunction in this inherited disorder, likely stemming from a combination of pituitary and gonadal iron overload. Therefore, the term Thalassemia-Associated Mixed Hypogonadism (TAMH) may encapsulate the key features of this endocrine complication in thalassemia: its direct association with the disease and the combined central and peripheral factors contributing to its development. This term could help in better defining and understanding the specific nature of hypogonadism in this population, potentially leading to tailored diagnostic and therapeutic approaches.

Specific characteristics of continuous glucose monitoring have identified the “transfusion-dependent prediabetes”, demonstrating that endocrinopathies in TDT subjects present peculiar characteristics that are never entirely comparable to already known clinical entities [51].

Therefore, endocrine complications necessitate a holistic approach to diagnosis and management, and the specific challenges and potential cardiovascular implications of hypogonadism in the context of TDT patients need to be considered. Further research is warranted to fully understand the underlying mechanisms and to develop targeted interventions to improve the quality of life and long-term outcomes for these patients.

## Data Availability

The data that supports the findings of this study are available from the corresponding author upon reasonable request.
