# Correction: Thalassemia-Associated mixed hypogonadism (TAMH): unraveling a unique endocrine pattern and its impact on cardiovascular risk

**DOI:** 10.1007/s12020-025-04457-8

**Published:** 2025-10-23

**Authors:** Maddalena Casale, Martina Errico, Francesca Allosso, Lucia Digitale Selvaggio, Graziella Grande, Claudia Di Ludovico, Raffaele Navarra, Domenico Roberti, Maria Maddalena Marrapodi, Silverio Perrotta, Daniela Pasquali

**Affiliations:** 1 Department of Woman, Child and General and Specialized Surgery, University of Campania L. Vanvitelli, Naples, Italy; 2 Department of Advanced Medical and Surgical Sciences, University of Campania L. Vanvitelli, Naples, Italy

For completeness and transparency, the old incorrect version and the corrected version of Tables are displayed below

Incorrect Table 1:

**Table 1:** Characteristics of the population examined

CV risk and metabolic profile Mean (SD) or *N* (%)	NH-TDT	H-TDT	HH-C	*p*-value^1^	*p*-value^2^
Progetto Cuore score^3^	0.77 (1.04)	0.87 (0.83)	4.12 (7.86)	0.13	0.6
Framingham score^4^	3.2 (3.5)	4.0 (2.9)	8.0 (12.0)	0.046*	0.2
Total cholesterol (mg/dl)	113 (26)	136 (27)	185 (43)	0.004*	< 0.001*
HDL-c (mg/dl)	35 (14)	39 (11)	51 (13)	0.15	< 0.001*
Triglycerides (mg/dl)	93 (42)	106 (62.1)	118 (58.6)	0.907	0.446
LDL-c (mg/dl)	66 (23.8)	78 (32.3)	107 (38)	0.116	0.007*
PAs (mmHg)	112 (10)	107 (9)	119 (9)	0.083	< 0.001*
PAd (mmHg)	61 (7.7)	61 (9.9)	74 (10.2)	0.814	< 0.001*

^1^p-value NH-TDT vs. H-TDT

^2^ p-value H-TDT vs. HH-C

^3^ Low risk < 3%; intermediate risk 3–20%; high risk > 20%

^4^ Low risk < 10%; intermediate risk 10–19%; high risk > 20%

The table summarizes the main clinical and demographic features of the three patient groups: non-hypogonadal thalassemic patients (NH-TDT), hypogonadal thalassemic patients (H-TDT), and hypogonadal non-thalassemic patients (HH-C). For each group, data are presented regarding mean age, sex distribution, and the presence of diabetes and smoking habits. In hypogonadal patients (H-TDT and HH-C), adherence to hormone replacement therapy was also assessed. The presence of splenectomy was recorded only in thalassemic patients (TDT and H-TDT). Continuous variables are expressed as mean (standard deviation), and categorical variables as number (percentage).

Age, a continuous variable, was analyzed using the Wilcoxon rank sum test due to non-normality. Categorical variables were compared using Pearson’s chi-squared test, except when low frequencies were present, in which case Fisher’s exact test was applied.

Corrected Table 1:

**Table 1:** Characteristics of the population examined

Characteristics Mean (SD) or *N* (%)	NH-TDT	H-TDT	HH-C	*p*-value^1^	*p*-value^2^
N.	31	22	29		
Age (years)	37 (12)	41 (9)	38 (20)	0.2	0.1
F	17 (55%)	14 (64%)	9 (31%)	0.5	0.02
M	14 (45%)	8 (36%)	20 (69%)		
Compliance to therapy	-	17 (77%)	28 (97%)	-	0.073
Diabetes	0 (0%)	3 (14%)	0 (0%)	0.066	0.074
Smoking	4 (13%)	8 (36%)	7 (25%)	0.5	0.4
Splenectomy	12 (39%)	17 (77%)	-	0.005*	-

The table summarizes the main clinical and demographic features of the three patient groups: non-hypogonadal thalassemic patients (NH-TDT), hypogonadal thalassemic patients (H-TDT), and hypogonadal non-thalassemic patients (HH-C). For each group, data are presented regarding mean age, sex distribution, and the presence of diabetes and smoking habits. In hypogonadal patients (H-TDT and HH-C), adherence to hormone replacement therapy was also assessed. The presence of splenectomy was recorded only in thalassemic patients (TDT and H-TDT). Continuous variables are expressed as mean (standard deviation), and categorical variables as number (percentage).

Age, a continuous variable, was analyzed using the Wilcoxon rank sum test due to non-normality. Categorical variables were compared using Pearson’s chi-squared test, except when low frequencies were present, in which case Fisher’s exact test was applied

Incorrect Table 2:

**Table 2:** Hormonal assessment

Characteristics Mean (SD) or *N* (%)	NH-TDT	H-TDT	HH-C	*p*-value^1^	*p*-value^2^
N.	31	22	29		
Age (years)	37 (12)	41 (9)	38 (20)	0.2	0.1
F	17 (55%)	14 (64%)	9 (31%)	0.5	0.02
M	14 (45%)	8 (36%)	20 (69%)		
Compliance to therapy	-	17 (77%)	28 (97%)	-	0.073
Diabetes	0 (0%)	3 (14%)	0 (0%)	0.066	0.074
Smoking	4 (13%)	8 (36%)	7 (25%)	0.5	0.4
Splenectomy	12 (39%)	17 (77%)	-	0.005*	-

The table presents serum levels of LH and FSH in all patients, as well as estradiol levels in women and testosterone levels in men in NH-TDT, H-TDT, and HH-C groups. Hormone values are expressed as mean (standard deviation). Variables were analyzed using the Wilcoxon rank sum test. When sample sizes were small or distributions included tied values, the exact version of the test was applied

Corrected Table 2:

**Table 2:** Hormonal assessment

Hormonal levels Mean (SD)	NH-TDT	H-TDT	HH-C	*p*-value^1^	*p*-value^2^
Estradiol Female (pmol/l)	209.25 (168.87)	69.75 (58.74)	37.08 (7.23)	0.005*	0.7
Testosterone Male (nmol/l)	19.78 (6.35)	5.66 (2.71)	1.98 (0.86)	< 0.001*	0.01*
FSH (IU/L)	12 (17.0)	4.2 (4.0)	1.8 (2.3)	0.006*	0.003*
LH (IU/L)	6.4 (4.7)	2.8 (2.9)	0.9 (1.1)	< 0.001*	0.001*

The table presents serum levels of LH and FSH in all patients, as well as estradiol levels in women and testosterone levels in men in NH-TDT, H-TDT, and HH-C groups. Hormone values are expressed as mean (standard deviation). Variables were analyzed using the Wilcoxon rank sum test. When sample sizes were small or distributions included tied values, the exact version of the test was applied

Incorrect Table 3:

**Table 3:** CV risk and lipid profile

Hormonal levels Mean (SD)	NH-TDT	H-TDT	HH-C	*p*-value^1^	*p*-value^2^
Estradiol Female (pmol/l)	209.25 (168.87)	69.75 (58.74)	37.08 (7.23)	0.005*	0.7
Testosterone Male (nmol/l)	19.78 (6.35)	5.66 (2.71)	1.98 (0.86)	< 0.001*	0.01*
FSH (IU/L)	12 (17.0)	4.2 (4.0)	1.8 (2.3)	0.006*	0.003*
LH (IU/L)	6.4 (4.7)	2.8 (2.9)	0.9 (1.1)	< 0.001*	0.001*

The table presents total cholesterol, HDL cholesterol, LDL cholesterol, triglycerides, systolic blood pressure (PAs), diastolic blood pressure (PAd) and CV risk scores calculated using the progetto Cuore and Framingham models in NH-TDT, H-TDT, and HH-C groups. All values are reported as mean (standard deviation). Variables were analyzed using the Wilcoxon rank sum test. When sample sizes were small or distributions included tied values, the exact version of the test was applied

Corrected Table 3:

**Table 3:** CV risk and lipid profile

CV risk and metabolic profile Mean (SD) or *N* (%)	NH-TDT	H-TDT	HH-C	*p*-value^1^	*p*-value^2^
Progetto Cuore score^3^	0.77 (1.04)	0.87 (0.83)	4.12 (7.86)	0.13	0.6
Framingham score^4^	3.2 (3.5)	4.0 (2.9)	8.0 (12.0)	0.046*	0.2
Total cholesterol (mg/dl)	113 (26)	136 (27)	185 (43)	0.004*	< 0.001*
HDL-c (mg/dl)	35 (14)	39 (11)	51 (13)	0.15	< 0.001*
Triglycerides (mg/dl)	93 (42)	106 (62.1)	118 (58.6)	0.907	0.446
LDL-c (mg/dl)	66 (23.8)	78 (32.3)	107 (38)	0.116	0.007*
PAs (mmHg)	112 (10)	107 (9)	119 (9)	0.083	< 0.001*
PAd (mmHg)	61 (7.7)	61 (9.9)	74 (10.2)	0.814	< 0.001*

^1^p-value NH-TDT vs. H-TDT

^2^ p-value H-TDT vs. HH-C

^3^ Low risk < 3%; intermediate risk 3–20%; high risk > 20%

^4^ Low risk < 10%; intermediate risk 10–19%; high risk > 20%

The table presents total cholesterol, HDL cholesterol, LDL cholesterol, triglycerides, systolic blood pressure (PAs), diastolic blood pressure (PAd) and CV risk scores calculated using the progetto Cuore and Framingham models in NH-TDT, H-TDT, and HH-C groups. All values are reported as mean (standard deviation). Variables were analyzed using the Wilcoxon rank sum test. When sample sizes were small or distributions included tied values, the exact version of the test was applied

The original article has been corrected.

